# Hemophilia B acquired after cadaveric liver transplantation: a case report

**DOI:** 10.1093/jscr/rjac393

**Published:** 2022-09-05

**Authors:** Larissa Machado e Silva Gomide, Viktoria Weihermann, Isabella Corrêa de Oliveira, Maria Alice Zarate Nissel, Igor Raphael Mathias Valejo, Lucas da Silva Wolff, Alan Junior de Aguiar, Dunia Verona, Ygor Degraf, Julianna Storace de Carvalho Arouca, João Paulo Barros Sanches, Rodrigo Rezende Silva Cabral, Katia Cristina Kampa, Alexandre Coutinho Teixeira de Freitas, Nertan Luiz Tefili

**Affiliations:** Department of Gastrointestinal Surgery and Liver Transplantation, Clinical Hospital of the Federal University of Paraná, Curitiba, PR, Brasil; Department of General Surgery, Clinical Hospital of the Federal University of Paraná, Curitiba, PR, Brasil; Department of General Surgery, Clinical Hospital of the Federal University of Paraná, Curitiba, PR, Brasil; Department of General Surgery, Clinical Hospital of the Federal University of Paraná, Curitiba, PR, Brasil; Department of General Surgery, Clinical Hospital of the Federal University of Paraná, Curitiba, PR, Brasil; Department of General Surgery, Clinical Hospital of the Federal University of Paraná, Curitiba, PR, Brasil; Department of Gastrointestinal Surgery and Liver Transplantation, Clinical Hospital of the Federal University of Paraná, Curitiba, PR, Brasil; Department of General Surgery, Clinical Hospital of the Federal University of Paraná, Curitiba, PR, Brasil; Department of General Surgery, Clinical Hospital of the Federal University of Paraná, Curitiba, PR, Brasil; Department of General Surgery, Clinical Hospital of the Federal University of Paraná, Curitiba, PR, Brasil; Department of General Surgery, Clinical Hospital of the Federal University of Paraná, Curitiba, PR, Brasil; Department of General Surgery, Clinical Hospital of the Federal University of Paraná, Curitiba, PR, Brasil; Department of Gastrointestinal Surgery and Liver Transplantation, Clinical Hospital of the Federal University of Paraná, Curitiba, PR, Brasil; Department of Gastrointestinal Surgery and Liver Transplantation, Clinical Hospital of the Federal University of Paraná, Curitiba, PR, Brasil; Department of General Surgery, Clinical Hospital of the Federal University of Paraná, Curitiba, PR, Brasil; Department of Gastrointestinal Surgery and Liver Transplantation, São Vicente Hospital, Curitiba, PR, Brasil

## Abstract

Hemophilia B is a recessive hereditary disease, and manifestations result from coagulation factor IX deficiency. Although improbable, as factor IX is produced exclusively in the liver, the possibility of developing the disease after transplantation represents an infrequent but potentially morbid complication. Standard laboratory tests may be insufficient to determine the probability of transmission of this pathology. This report describes the case of a patient who developed hemophilia B after liver transplantation whose donor had no prior knowledge of the disease.

## INTRODUCTION

Hemophilia B is a recessive hereditary disease, linked to the X chromosome that compromises one in every 15–30 000 male births [1]. Manifestations result from coagulation factor IX deficiency. Rare cases of acquired hemophilia B after liver transplantation are described in the literature. As factor IX is produced exclusively in the liver, there is a possibility of developing the disease after transplantation. We describe the case of a middle-aged patient who developed hemophilia B after orthotopic liver transplantation.

## CASE REPORT

A 46-year-old male patient, diagnosed with alcoholic liver cirrhosis and portal hypertension, without other comorbidities or previous surgeries, underwent cadaveric liver transplantation after 6 months of abstinence. The donor was a young patient victim of traumatic brain injury caused by a car accident. His medical history included tabagism, but no pathologies were known. Laboratory tests presented only anemia and mild thrombocytopenia, whereas serologic tests were all negative. The liver had a good macroscopic appearance during organ harvesting. The graft implantation was performed without events. Venous reconstruction was done through the Piggyback technique and cold ischemia time was around 6 h.

The patient evolved with graft dysfunction, presenting bilirubin elevation and altered coagulogram, which lead to episodes of massive bleeding requiring multiple reoperations. A total of 11 re-approaches were carried out to control hemostasis. Dialysis was necessary due to acute kidney injury and the patient’s need of prolonged ventilator support corroborated in the development of pulmonary sepsis. The condition was stabilized after 3 months and the patient was discharged.

During follow-up, it was observed normalization of the blood count and ProthrombinTime, but maintained alterations in the Activated Partial Thromboplastin Time (aPTT), bilirubin and canalicular enzymes tests. In a complementary investigation, measurement of coagulation factors was performed, identifying 4.1% activity of factor IX, confirming the diagnosis of hemophilia B.

## DISCUSSION

Hemophilia B, also called Christmas disease, is an inherited deficiency of factor IX, being a recessive disease linked to the X chromosome. To the best of our knowledge, this case represents the fourth reported liver transplantation transmission of hemophilia B [1–3]. Considering all the cases of hemophilia A and B liver transplantation transmission reported in the literature, the donors died after a traumatic event, most of them after intracranial hemorrhage and had no prior awareness of the disease [2, 3]. This was also observed in the present report. Cases with no family history are common, they are caused by a new mutation and represent 43% of hemophilia B patients [4]. An autoantibody-mediated form of acquired hemophilia B has also been described, although it is less common [5].

Hemophilias can be characterized as mild, moderate or severe, according to the activity level of the basal factor. Factor levels usually correlate with the degree of symptoms. It is considered severe when the factor activity is <1%, moderate when between 1 and 5% and mild when greater than 5% [6]. Our patient, despite multiple bleeding events, was classified as moderate.

Hemophilia is characterized by a prolonged time of activated partial thromboplastin. However, especially in the case of hemophilia B, individuals with moderate disease may present a normal aPTT. Furthermore, there are individuals in whom the disease may only become apparent when there is a significant event, such as trauma or surgery [7, 8]. This may explain the fact that our donor was not aware of his condition. Management of acute bleeding episodes includes factor IX-replacement, although this treatment is limited by the development of factor inhibitors in 25% of hemophiliacs.

The transmission of this disease by organ transplantation represents an infrequent but potentially morbid complication. A question that arises in this context is the need to screen for hemophilia in donors. The assessment of coagulation tests, such as the aPTT, is simple and inexpensive. However, as mentioned above, such exams may be normal even if the individual has moderate hemophilia. Also, critically ill patients commonly present altered coagulation tests for other causes than hemophilia. Standard laboratory tests may be insufficient to determine the probability of transmission of this pathology. Thus, a detailed anamnesis related to the personal history of bleeding and family history of diseases could be helpful to avoid this serious situation.
